# Population Size Estimation of Gay and Bisexual Men and Other Men Who Have Sex With Men Using Social Media-Based Platforms

**DOI:** 10.2196/publichealth.9321

**Published:** 2018-02-08

**Authors:** Stefan Baral, Rachael M Turner, Carrie E Lyons, Sean Howell, Brian Honermann, Alex Garner, Robert Hess III, Daouda Diouf, George Ayala, Patrick S Sullivan, Greg Millett

**Affiliations:** ^1^ Department of Epidemiology Johns Hopkins School of Public Health Baltimore, MD United States; ^2^ Hornet Gay Social Network San Francisco, CA United States; ^3^ Public Policy Office The Foundation for AIDS Research Washington, DC United States; ^4^ Enda-Santé Dakar Senegal; ^5^ The Global Forum for MSM and HIV Oakland, CA United States; ^6^ Department of Epidemiology Rollins School of Public Health Emory University Atlanta, GA United States

**Keywords:** AIDS, estimates, HIV, key populations, men who have sex with men, social media

## Abstract

**Background:**

Gay, bisexual, and other cisgender men who have sex with men (GBMSM) are disproportionately affected by the HIV pandemic. Traditionally, GBMSM have been deemed less relevant in HIV epidemics in low- and middle-income settings where HIV epidemics are more generalized. This is due (in part) to how important population size estimates regarding the number of individuals who identify as GBMSM are to informing the development and monitoring of HIV prevention, treatment, and care programs and coverage. However, pervasive stigma and criminalization of same-sex practices and relationships provide a challenging environment for population enumeration, and these factors have been associated with implausibly low or absent size estimates of GBMSM, thereby limiting knowledge about the dynamics of HIV transmission and the implementation of programs addressing GBMSM.

**Objective:**

This study leverages estimates of the number of members of a social app geared towards gay men (Hornet) and members of Facebook using self-reported relationship interests in men, men and women, and those with at least one reported same-sex interest. Results were categorized by country of residence to validate official size estimates of GBMSM in 13 countries across five continents.

**Methods:**

Data were collected through the Hornet Gay Social Network and by using an a priori determined framework to estimate the numbers of Facebook members with interests associated with GBMSM in South Africa, Ghana, Nigeria, Senegal, Côte d'Ivoire, Mauritania, The Gambia, Lebanon, Thailand, Malaysia, Brazil, Ukraine, and the United States. These estimates were compared with the most recent Joint United Nations Programme on HIV/AIDS (UNAIDS) and national estimates across 143 countries.

**Results:**

The estimates that leveraged social media apps for the number of GBMSM across countries are consistently far higher than official UNAIDS estimates. Using Facebook, it is also feasible to assess the numbers of GBMSM aged 13-17 years, which demonstrate similar proportions to those of older men. There is greater consistency in Facebook estimates of GBMSM compared to UNAIDS-reported estimates across countries.

**Conclusions:**

The ability to use social media for epidemiologic and HIV prevention, treatment, and care needs continues to improve. Here, a method leveraging different categories of same-sex interests on Facebook, combined with a specific gay-oriented app (Hornet), demonstrated significantly higher estimates than those officially reported. While there are biases in this approach, these data reinforce the need for multiple methods to be used to count the number of GBMSM (especially in more stigmatizing settings) to better inform mathematical models and the scale of HIV program coverage. Moreover, these estimates can inform programs for those aged 13-17 years; a group for which HIV incidence is the highest and HIV prevention program coverage, including the availability of pre-exposure prophylaxis (PrEP), is lowest. Taken together, these results highlight the potential for social media to provide comparable estimates of the number of GBMSM across a large range of countries, including some with no reported estimates.

## Introduction

Consensual sex between adult men remains stigmatized in much of the world. Same-sex practices and relationships are criminalized in over 70 countries, many of which are countries where the HIV epidemic is most generalized [1]. Given pervasive social stigma around the world and punitive laws specifically affecting gay, bisexual, and other cisgender men who have sex with men (GBMSM), it remains challenging to count the numbers of GBMSM to inform the content and scale of specific health programs, including HIV prevention, treatment, and care services [2,3]. The biological risks that predispose GBMSM to HIV infection are similar around the world; however, there has been an assumption that there are less GBMSM in countries with generalized epidemics. In turn, this view has supported a further assumption regarding the limited relevance of the HIV prevention needs of GBMSM in the broader HIV transmission dynamics in these countries [4].

There is no consensus on the optimal strategy of measuring the numbers of GBMSM. The limited consensus reflects challenges in enumerating a diverse group of men comprising different sexual orientations and sexual practices over time. Specifically, there are GBMSM who identify as gay, bisexual, straight, or do not identify with a particular sexuality at all. There are those that only have sex with men, or both men and women, and those that report sexual practices with people along the gender continuum [5]. Moreover, there are those that only have sexual attraction to other men; however, they have not had sex, or have had certain forms of sex, but not penetrative anal sex with men. Finally, there is often conflation of sexual and gender identities, which complicates the enumeration of cisgender GBMSM because transgender women who have sex with men are sometimes (inappropriately) included within these counts [6,7]. These challenges are compounded by a pervasive stigma of homosexuality, and by the lack of consensus about the optimal questions and measures to enumerate men who have health risks associated with their sexual behaviors [8].

Population counts of GBMSM have included both in-person assessments, digital assessments, or surveys. In the United Kingdom, the Annual Population Survey has been used to estimate the numbers of GBMSM in the population, and multiple nationally representative samples have been leveraged in the United States to generate estimates of population size [9]. However, in most settings, including the United States, there is a greater reliance on digital strategies given that sexual identity is not included on the United States national census. Small area estimation methods have also been used for estimating GBMSM population sizes at smaller geographic levels within the United States [10]. In the United Kingdom in 2016, approximately 2.3% of men identified as gay or bisexual, whereas a Gallup Poll in the United States noted that approximately 4.1% of all people identified as lesbian, gay, bisexual, or transgender (LGBT), representing approximately 10 million people [11]. Not all national estimates of population size of GBMSM are from higher income settings. For example, investigators from the Other Foundation in South Africa reported that an estimated 530,000 people identified as gay, bisexual, or gender nonconforming [12].

In settings without methodologically sound national surveys, which represents most countries of the world, there is a great reliance on population size estimation strategies to assess the numbers of GBMSM. However, these approaches are collectively more difficult to implement in countries with stigmatizing settings where homosexuality may be criminalized, and where political will or leadership is absent. This issue reinforces a data paradox: we know the least about the numbers and social experiences of the most vulnerable communities of GBMSM in settings deemed the most hostile [13,14]. Additionally, high HIV service coverage goals may disincentivize accurate size estimates for countries with implausibly low or absent population estimates [15].

The normative agency responsible for providing estimates of population size to inform mathematical models of HIV transmission is the Joint United Nations Programme on HIV/AIDS (UNAIDS). Given the lack of national estimates of GBMSM, UNAIDS relies on United Nations member states to submit data on population size estimates [16]. These estimates have significant variability in the quality of the underlying studies and representativeness. Ultimately, many countries do not include any estimates of the numbers of GBMSM. In the absence of population estimates, especially in more generalized HIV epidemic settings, dedicated HIV prevention, treatment, and care programs for GBMSM remain limited or missing altogether. This lack of adequate data and resulting resource allocation might account for the increased mortality and morbidity among men living with HIV in countries with generalized HIV epidemics [17]. For example, in Tanzania in October of 2017, people working with organizations aimed at addressing the HIV prevention and treatment needs of GBMSM were arrested and then deported, highlighting the inherent challenges of understanding and ultimately addressing HIV risks among GBMSM [18].

Given the need to reliably estimate the proportion of the male population who do not identify as heterosexual, or who are not exclusively behaviorally heterosexual, this study compares UNAIDS estimates (where available) to estimates of members of Hornet (a social app geared towards gay men [19]) and Facebook members with specific interests associated with GBMSM in 13 countries across five continents.

## Methods

The overarching goal of this study was to assess the utility of using data on users of mobile phone apps and social media communities to obtain estimates of GBMSM population sizes. These estimates of GBMSM obtained from a gay social network app and Facebook users were compared to the most recent officially reported UNAIDS [20] and national estimates [10,21] across 13 countries. A selection of countries with geographic representation, along with areas with significant investment from the United States President’s Emergency Plan for AIDS Relief (PEPFAR) and the Global Fund for AIDS, Tuberculosis, and Malaria were included. These countries included seven from Sub-Saharan Africa (South Africa, Ghana, Nigeria, Senegal, Côte d'Ivoire, Mauritania, and The Gambia), one from the Middle East and North Africa (Lebanon), two from Asia (Thailand and Malaysia), one from South America (Brazil), one from Europe (Ukraine), and the United States. The number of unique active gay app users in 2015 who resided in each country were provided by the Hornet Gay Social Network (Hornet) [19].

Detailed targeting of unique users within Facebook’s Advertising (Ads) Manager was utilized to estimate the number of men who are interested in men (MIM), men who are interested in men or men and women (MIMW), and those men with at least one reported same-sex interest (MSSI) by country of residence (Multimedia Appendix 1,Multimedia Appendix 2,Multimedia Appendix 3). Facebook’s Ads Manager is structured around four domains that can be used to stratify users and produce size estimates: (1) location, (2) demographics, (3) interests, and (4) behaviors [22]. Facebook’s intelligent analytics engine can track online behavior (on and off Facebook) and code the user’s behavior into interest and behavior categories that appear in the Ads Manager; this is done based on: (1) what people share on their timelines; (2) apps they use; (3) ads they click on; (4) pages they engage with; (5) activities people engage in on and off Facebook related to things like their device usage, purchase behaviors or intents, and travel preferences; (6) demographics like age, gender, and location; and (7) the mobile device they use and the speed of their network connection [23]. Specifically, Facebook defines these variables as *interests* and *behaviors* [24]. Interests are used by marketers to reach specific audiences by looking at people’s interests, activities, the pages and posts they like, posts and comments they make, and closely related topics. Behaviors are used to facilitate targeting based on purchase behaviors, device usage, and other activities. While it is feasible to use both interests and behaviors to assess population size, this study used interests. Specifically, the following keywords were used to identify same-sex interests: “bisexual,” “gay,” “gender,” “homosexual,” “LGBT,” “pride,” “same-sex,” and “trans.” Related interests were also suggested by Facebook’s Ads Manager based on the aforementioned search terms. Identified same-sex interests were subsequently assigned to one of eight thematic groups, representing men who have expressed an interest in (or liked pages related to) at least one included same-sex interest (Multimedia Appendix 4). Only same-sex interests endorsed by at least 100,000 Facebook users across all of Facebook were included in the analysis. No ads were created. During the process of planning advertisements, the Facebook Ads Manager interface displays an estimated number of users in the geographic area with those interests. This estimate was used to capture the potential size of each country-specific audience meeting specified criteria (ie, the number of adult MIM, MIMW, and MSSI Facebook users who may be exposed to the ad per country). UNAIDS GBMSM population size estimation methodology has been reported elsewhere [16].

## Results

A comparison of GBMSM estimates from UNAIDS, Hornet, and Facebook is presented in Table 1 (see Multimedia Appendix 5 for more details). Recent UNAIDS and national estimates are less than those reported by active Hornet app users in Brazil, Thailand, and Ukraine, with differences in estimates of 1,018,520, 603,942, and 66,670, respectively. UNAIDS and national estimates of GBMSM are less than all three Facebook estimates categories (MSSI, MIMW, MIM) in Malaysia, Ghana, Nigeria, Lebanon, Senegal, Côte d'Ivoire, Mauritania, and The Gambia. UNAIDS and national estimates are less than the reported numbers of MSSI, MIMW, and MIM in 13, 9, and 8 selected countries, respectively.

The number of adult Facebook users identified in the eight same-sex interest categories, including *Entertainment/Media*, *Gay*, *Gender*, *LGBT*, *Pride*, *Relationships*, *Sexuality*, and *Trans*, is presented in Table 2 (see Multimedia Appendix 6 for more details), with all interest categories available in Multimedia Appendix 4. The proportions of men with Facebook that identified same-sex interest categories among the overall Facebook estimate of males >18 years of age is also presented. The proportion of men reporting a same-sex interest within the category of *Relationships* ranged from 1.6% in Ukraine to 25.5% in Nigeria, and those reporting interests categorized as being related to being *Gay* ranged from <0.5% in The Gambia to 9.4% in Ukraine.

The age distribution of Facebook users identified as MSSI, MIMW, and MIM is presented in Multimedia Appendix 7. The proportions of MSSI, MIMW, and MIM among the overall Facebook estimates of males by country is also presented.

The proportion of males aged 13-17 years reporting same-sex interests ranged from 4.7% in Côte d'Ivoire to 21.2% in the United States, and estimated MIM in the same age group ranged from <0.2% in Ukraine to 3.2% in Malaysia. In several countries, Facebook estimates of one or two age cohorts alone exceeded the UNAIDS total population estimate of GBMSM for the respective country.

**Table 1:** Comparison of GBMSM^a^ estimates from UNAIDS^b^, Hornet, and Facebook. MIM: men (>18 years of age) interested in men ; MIMW: men (>18 years of age) interested in men or men and women; MSSI: men (>18 years of age) with at least one reported same-sex interest.

Country	UNAIDS estimate^b^	Facebook estimates				Differences (UNAIDS minus indicated estimate)			
		MSSI	MIMW	MIM	Hornet Users	MSSI	MIMW	MIM	Hornet
United States^c^	4,503,080	17,000,000	1,500,000	740,000	2,482,802	(–12,496,920)	3,003,080	3,763,080	2,020,278
Brazil	2,037,741	9,900,000	510,000	240,000	3,056,261	(–7,862,259)	1,527,741	1,797,741	(–1,018,520)
South Africa^d^	654,979	870,000	430,000	61,000	38,013	(–215,021)	224,979	593,979	616,966
Thailand	571,000	3,600,000	760,000	290,000	1,174,942	(–3,029,000)	(–189,000)	281,000	(–603,942)
Ukraine	176,000	620,000	38,000	15,000	242,670	(–444,000)	138,000	161,000	(–66,670)
Malaysia	170,000	3,000,000	2,100,000	290,000	311,472	(–2,830,000)	(–1,930,000)	(–120,000)	(–141,472)
Ghana	30,579	700,000	550,000	42,000	31,165	(–v669,421)	(–519,421)	(–11,421)	(–586)
Nigeria	29,470	3,300,000	2,800,000	180,000	14,793	(–3,270,530)	(–2,770,530)	(–150,530)	14,677
Lebanon	3114	260,000	68,000	29,000	25,916	(–256,886)	(–64,886)	(–25,886)	(–22,802)
Senegal	1840	260,000	190,000	31,000	2870	(–258,160)	(–188,160)	(–29,160)	(–1030)
Côte d’Ivoire	1343	340,000	220,000	23,000	3575	(–338,657)	(–218,657)	(–21,657)	(–2232)
Mauritania	160	47,000	31,000	7300	177	(–46,840)	(–30,840)	(–7140)	(–17)
The Gambia	150	34,000	20,000	1800	194	(–33,850)	(–19,850)	(–1650)	(–44)

^a^GBMSM: Gay, bisexual, and other cisgender men who have sex with men.

^b^UNAIDS: Joint United Nations Programme on HIV/AIDS. Reported GBMSM size estimate from the UNAIDS AIDS Info Online Database [20] unless otherwise specified.

^c^United States GBMSM size estimate from Grey et al 2016 [10].

^d^South Africa GBMSM size estimate from 2017 PEPFAR COP [21].

**Table 2:** Distribution of Facebook-identified same-sex interests by country. Reported in thousands, n (%).

Country	Entertainment/ Media	Gay	Gender	LGBT	Pride	Relationships	Sexuality	Trans
United States	7100 (6.8)	4500 (4.3)	2800 (2.7)	11,000 (10.6)	5600 (5.4)	3800 (3.7)	4900 (4.7)	23 (0.0)
Brazil	2900 (5.3)	2200 (4.0)	710 (1.3)	7400 (13.5)	3500 (6.4)	4700 (8.5)	2800 (5.1)	110 (0.2)
South Africa	150 (2.0)	100 (1.4)	51 (0.7)	330 (4.5)	140 (1.9)	480 (6.5)	130 (1.8)	<1 (0.0)
Thailand	530 (2.3)	400 (1.7)	110 (0.5)	2200 (9.6)	390 (1.7)	920 (4.0)	1200 (5.2)	6.6 (0.0)
Ukraine	470 (9.8)	450 (9.4)	14 (0.3)	170 (3.5)	71 (1.5)	79 (1.6)	77 (1.6)	<1 (0.0)
Malaysia	350 (2.7)	260 (2.0)	89 (0.7)	780 (6.0)	320 (2.5)	2200 (16.9)	400 (3.1)	6.2 (0.0)
Ghana	55 (1.7)	24 (0.8)	30 (0.9)	130 (4.1)	43 (1.3)	580 (18.1)	50 (1.6)	<1 (0.0)
Nigeria	220 (2.0)	110 (1.0)	81 (0.7)	470 (4.3)	140 (1.3)	2800 (25.5)	210 (1.9)	1.9 (0.0)
Lebanon	59 (3.0)	55 (2.8)	18 (0.9)	100 (5.0)	50 (2.5)	83 (4.2)	84 (4.2)	<1 (<0.1)
Senegal	16 (0.9)	6.6 (0.4)	18 (1.1)	59 (3.5)	13 (0.8)	190 (11.2)	13 (0.8)	<1 (<0.1)
Côte d’Ivoire	16 (0.6)	8.4 (0.3)	13 (0.5)	72 (2.9)	57 (2.3)	240 (9.6)	32 (1.3)	<1 (0.0)
Mauritania	3.5 (0.9)	2.3 (0.6)	6.9 (1.9)	8.6 (2.3)	2.5 (0.7)	32 (8.6)	3.9 (1.1)	n/a
The Gambia	1.3 (0.7)	<1 (<0.5)	1.3 (0.7)	13 (6.5)	1.3 (0.7)	21 (10.5)	12 (6.0)	n/a

## Discussion

### Principal Findings

Comparisons of UNAIDS GBMSM size estimates to numbers of users on a gay-oriented app and Facebook users with same-sex interests suggest that UNAIDS data may significantly underestimate GBMSM populations to varying extents across all countries. Although there are several limitations to our approach, there is a clear signal towards higher estimates of GBMSM when digital approaches are utilized. While several studies have focused on the role dating apps and websites play in risk-taking behaviors among GBMSM [25], only recently has there been interest in leveraging apps to serve the HIV prevention, treatment, and care needs among GBMSM. Our data suggest that same-sex dating apps and social media networks are promising data sources for designing population estimates and programmatic targets for GBMSM.

The usage of the Internet is becoming increasingly normalized throughout the world, and this trend will only continue. Empirical and market-research data, generally derived from higher income settings, demonstrate that GBMSM are high utilizers of the Internet, often using the Internet to find partners, given limited venues and significant social stigma [26-30]. Moreover, GBMSM who find partners online may be at higher risk of HIV acquisition and transmission [31]. This trend towards online spaces has largely been attributed to stigma that same-sex behaviors face and the confidentiality that is afforded in online spaces [32-34], which enable users to more accurately report their attraction (or in some instances, trace their behavior) and eliminate biases in self-reported data. Although these studies were predominantly completed in higher income settings, similar results have been observed among GBMSM in Southern Africa and Nigeria [35,36]. Given the significant usage of the Internet, online spaces likely represent an important approach for collecting same-sex attraction and behavioral data, especially in more stigmatizing settings. However, these same spaces also represent a currently underutilized approach to better address the HIV prevention and treatment needs of GBMSM [37].

Little is known about the needs of GBMSM under 18 years of age, because these young GBMSM are often not allowed to enroll in studies or seek services. However, most men have their first sexual encounters with other men before the age of 18, and HIV incidence has been shown to be high among young GBMSM across settings [38,39]. Moreover, men under the age of 18 are generally not included in surveys or HIV prevention, treatment, and care programs, given the challenges in achieving consent [40]. Given that younger GBMSM are more likely to leverage virtual spaces, as evidenced by these data, the use of digital data for size estimation represents a strategy to inform the numbers and HIV prevention, treatment, and care needs of young GBMSM [41]. While the legal challenges of consent remain, especially in settings where same-sex practices are criminalized, there is a clear need to scale up evidence-based and human rights-affirming HIV prevention strategies for younger GBMSM, including condoms, lubricants, and preexposure prophylaxis (PrEP).

### Limitations

There are several limitations in the methods and results presented here. Detailed information about how Facebook’s Ads Manager projects ad reach estimates–or population estimates for the intents and purposes of this article–is not public, and discrepancies have been identified between their projections and census data [42,43]. The definitions of men’s same-sex interests used here were derived by the authors and may be nonspecific, potentially including people interested in LGBT equality (allies). Moreover, the extent of specificity is likely subject to specific cultural contexts, thereby leading to overestimating the numbers of GBMSM with this metric. The metric used for Hornet was unique users with only one account allowed per device. While it is feasible to create multiple accounts on Facebook with unique email addresses, this likely represents a very low proportion of users. Although Internet usage is increasing rapidly, there is less access in many lower- and middle-income countries, which may underestimate the numbers of GBMSM. However, Internet access around the world continues to increase, especially due to the rapid increase in affordable smartphones, suggesting that the utility of social media-based estimates of population size will increase over time. Moreover, some of the respondents captured online in low- and middle-income countries may be expatriates rather than GBMSM from that country. The contribution of expatriates to these estimates is considered to be low, given the limited number of expatriates, and only people who noted the country as their country of residence were included. Additional research studying appropriate search strategies according to each social media platform, including large platforms not studied in these analyses, are required. Building collaborations with social media platforms may also facilitate improved estimates of population size along with insights into appropriate strategies to deliver interventions that leverage these platforms. Taken together, these data clearly suggest a significant discrepancy between size estimates of GBMSM reported by normative agencies and estimates from digital sources.

### Conclusions

Over four decades of the HIV pandemic, GBMSM have been well known to bear a disproportionate burden of HIV due to the biology of the virus, which is compounded by criminalization, intersectional stigma, discrimination, and violence. Deriving estimates of the numbers of people at risk of acquiring and living with HIV is complex, and other studies have highlighted these challenges for other populations. For GBMSM, deriving estimates is further complicated by hostile policy settings, where HIV epidemics are also characterized as *generalized*. Developing common methods of counting GBMSM, especially the use of central data collection with consistent approaches, provides an additional data source that is directly comparable across settings. Although these additional approaches have biases, they are complementary to the biases of existing methods. The approach presented here that leverages social media is imperfect, but is relatively low cost to implement and provides comparable estimates across a large range of countries, including some with no extant estimates. Triangulating multiple data sources (including social media) may facilitate optimal estimations of the numbers of GBMSM for program planning, evaluation, and estimates of HIV epidemic dynamics. These methods also allow for the estimation of numbers of GBMSM in settings where stigma and risks of violence are too great to even report in this paper [44]. As violence targeting GBMSM continues to escalate, traditional estimation of the numbers of GBMSM are increasingly problematic. The practice of pointing to no data (in this instance nonexistent GBMSM size estimates) to justify not funding or grossly underresourcing programs for GBMSM has long been identified by advocates, and should be challenged. Not doing so runs the risk of having evidence-based and human rights-affirming programs that address specific needs of GBMSM disappear. Ultimately, we cannot overstate the importance of understanding the characteristics and numbers of those most affected by HIV to truly achieve an AIDS-free generation.

## Appendix

Multimedia Appendix 1 Screenshot of detailed targeting within Facebook’s Ads Manager used to estimate the number of adult male residents of Malaysia who are interested in men (MIM).

Multimedia Appendix 2 Screenshot of detailed targeting within Facebook’s Ads Manager used to estimate the number of adult male residents of Malaysia who are interested in men or both men and women (MIMW).

Multimedia Appendix 3 Screenshot of detailed targeting within Facebook’s Ads Manager used to estimate the number of adult male residents of Malaysia who report at least one same-sex interest (MSSI).

Multimedia Appendix 4 Facebook identified same-sex interest categories included for MSSI.

Multimedia Appendix 5 Comparison of gay, bisexual, and other men who have sex with men (GBMSM) estimates from UNAIDS, Hornet, and Facebook — expanded Table 1.

Multimedia Appendix 6 Distribution of Facebook identified same-sex interests by country — expanded Table 2.

Multimedia Appendix 7 Age distribution of male Facebook users by country (proportion of males).

## Abbreviations

Ads: Advertisements
AIDS: acquired immunodeficiency syndrome
GBMSM: gay, bisexual, and other men who have sex with men
LGBT: lesbian, gay, bisexual, or transgender
MIM: men interested in men
MIMW: men interested in men or men and women
MSSI: men with at least one reported same-sex interest
NIH: National Institutes of Health
PEPFAR: President’s Emergency Plan for AIDS Relief
PrEP: pre-exposure prophylaxis
UNAIDS: Joint United Nations Programme on HIV/AIDS
