# Supplementary figures and images for: Population Size Estimation of Gay and Bisexual Men and Other Men Who Have Sex With Men Using Social Media-Based Platforms
Source: JMIR Public Health Surveill. 2018 Feb 8;4(1):e15. doi: 10.2196/publichealth.9321 (PMC5824103; doi:10.2196/publichealth.9321)

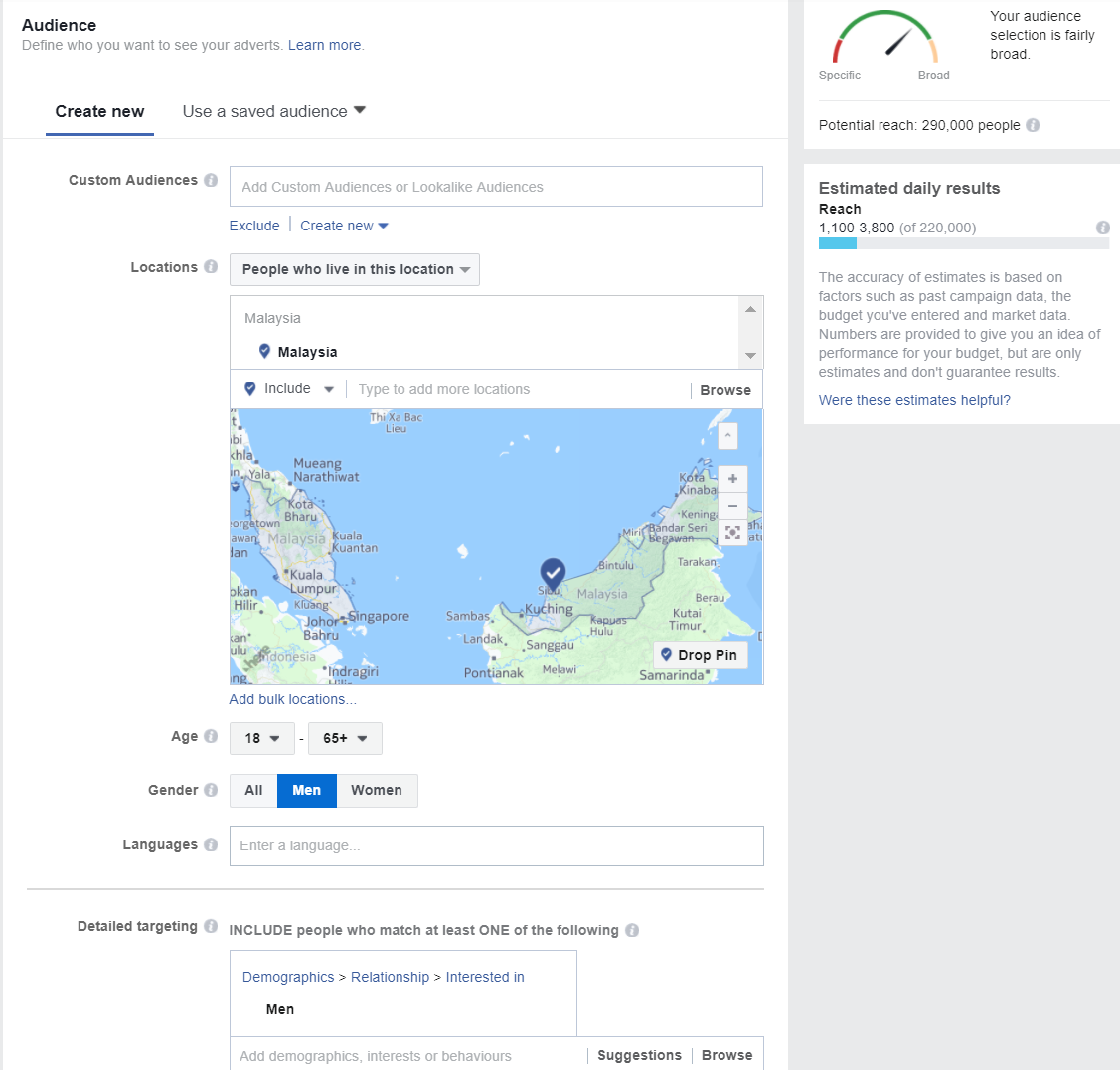

Supplement: Multimedia Appendix 1 [file publichealth_v4i1e15_app1.png]

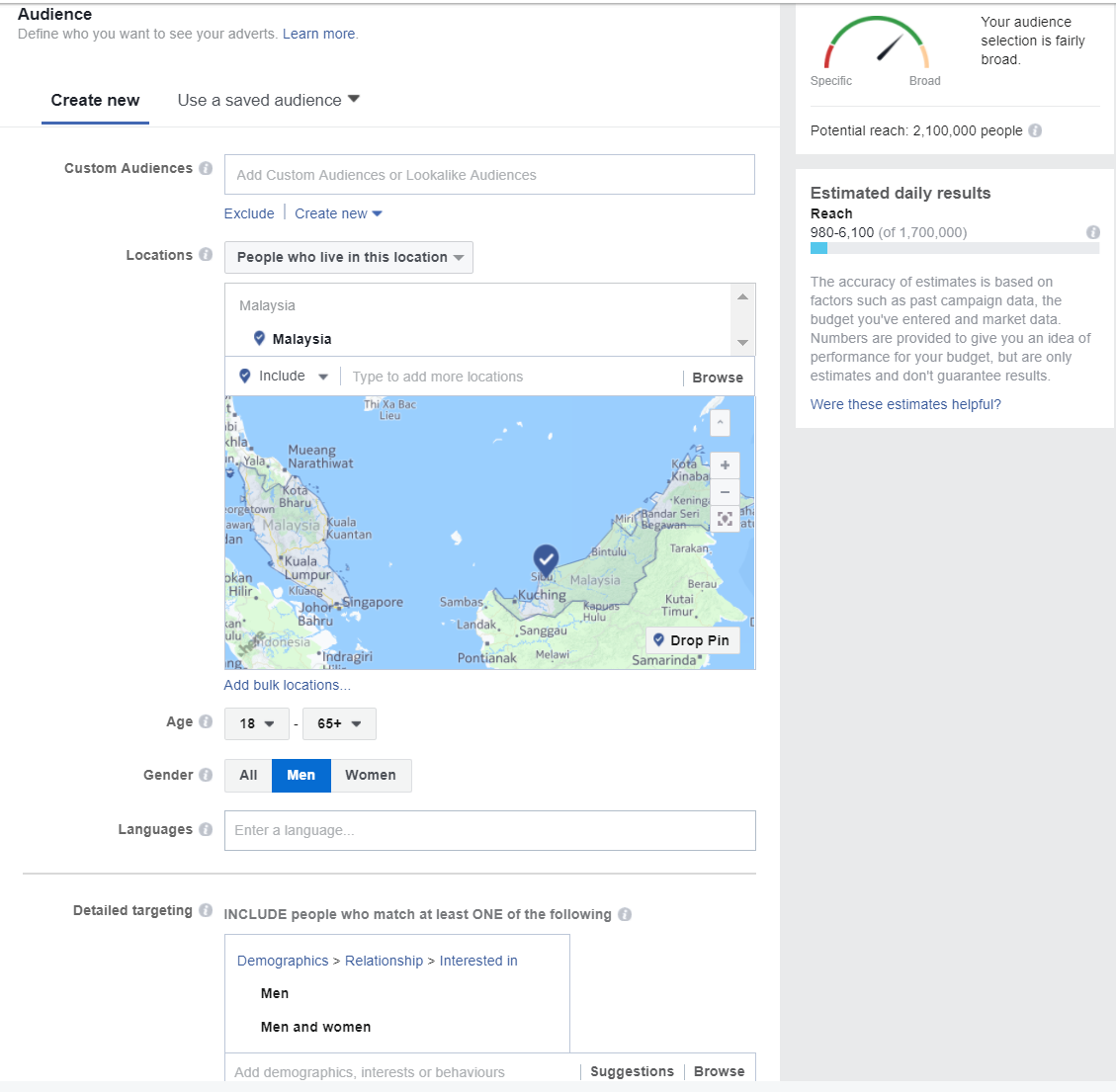

Supplement: Multimedia Appendix 2 [file publichealth_v4i1e15_app2.png]

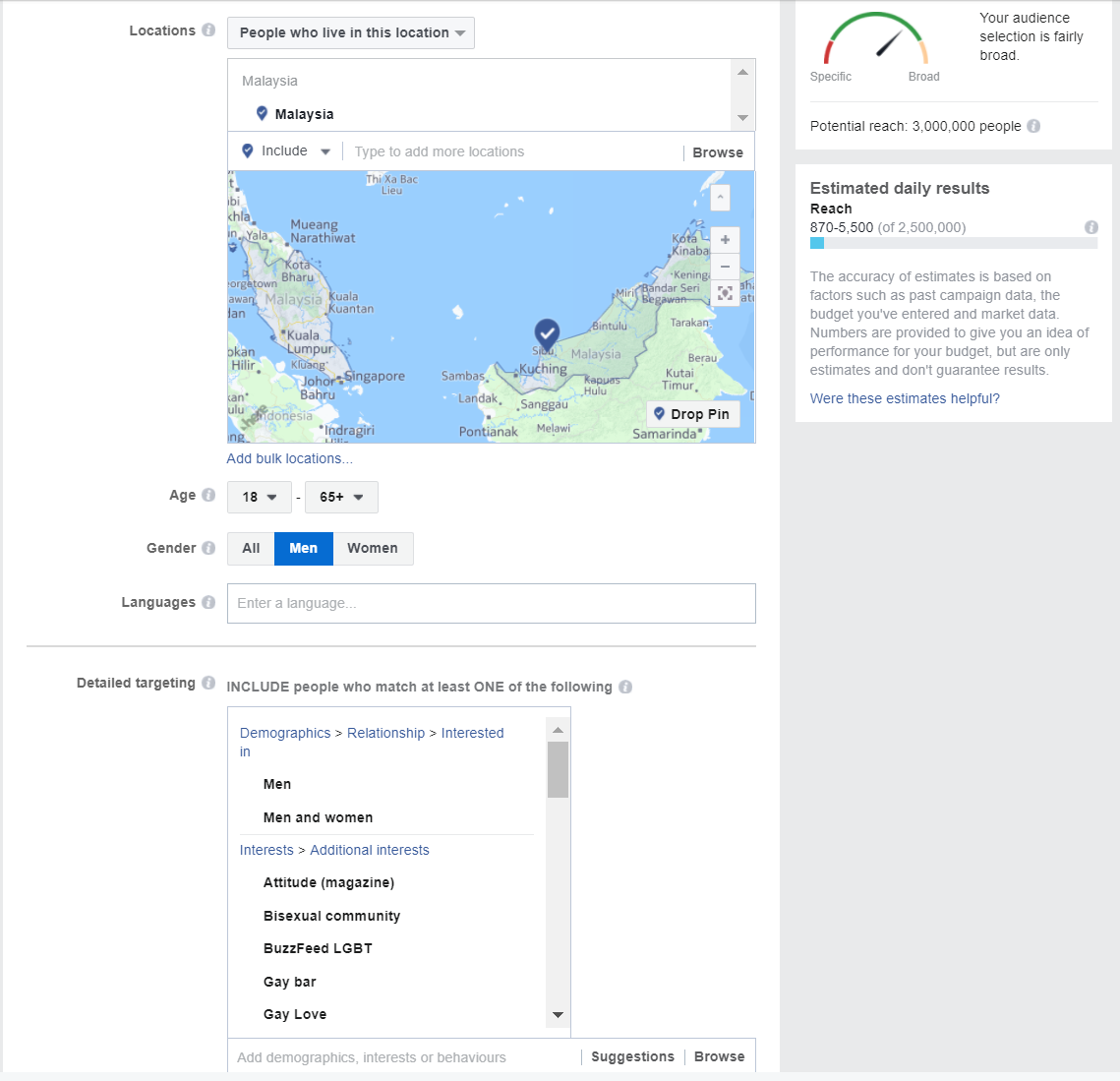

Supplement: Multimedia Appendix 3 [file publichealth_v4i1e15_app3.png]

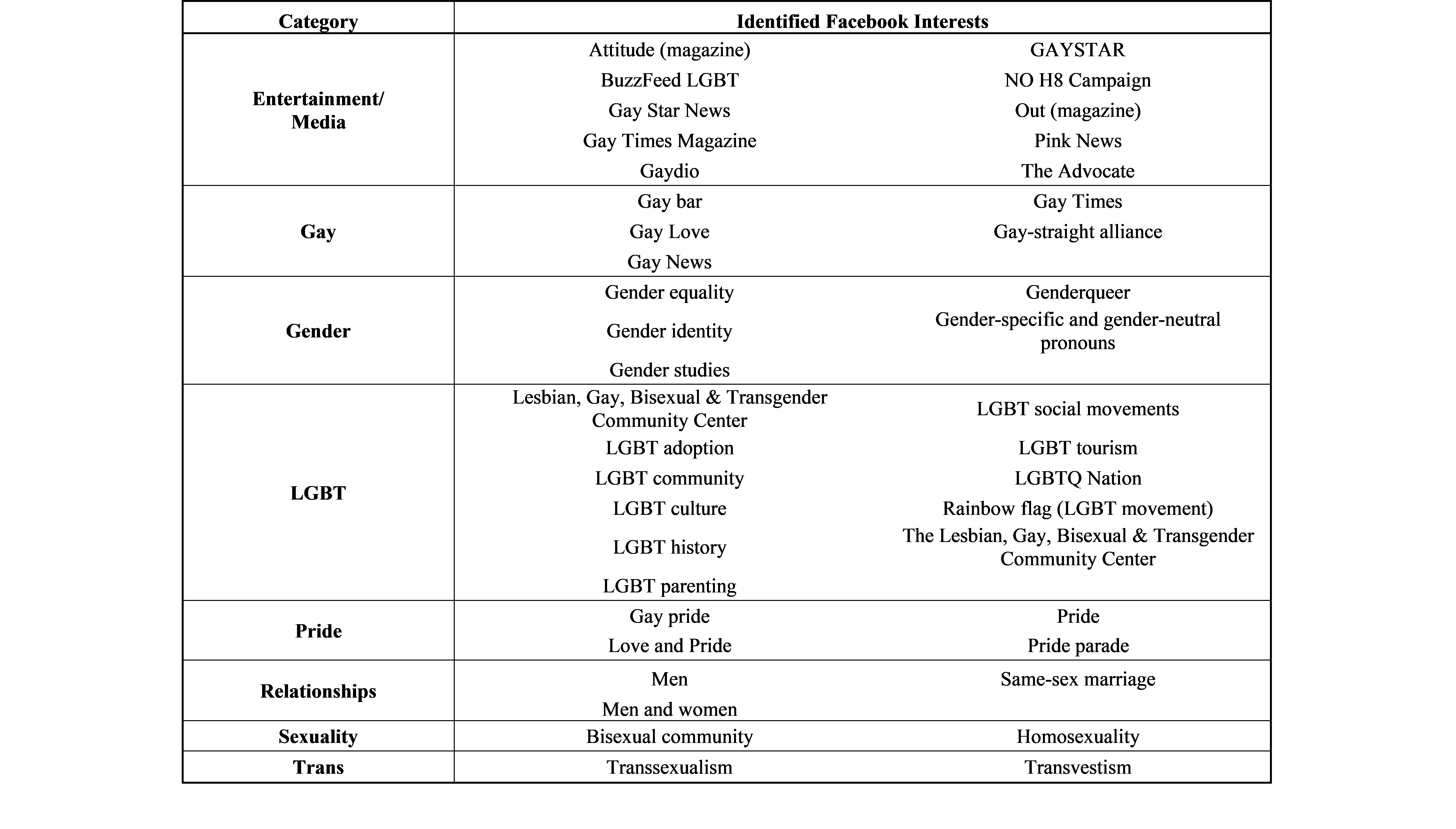

Supplement: Multimedia Appendix 4 [file publichealth_v4i1e15_app4.png]

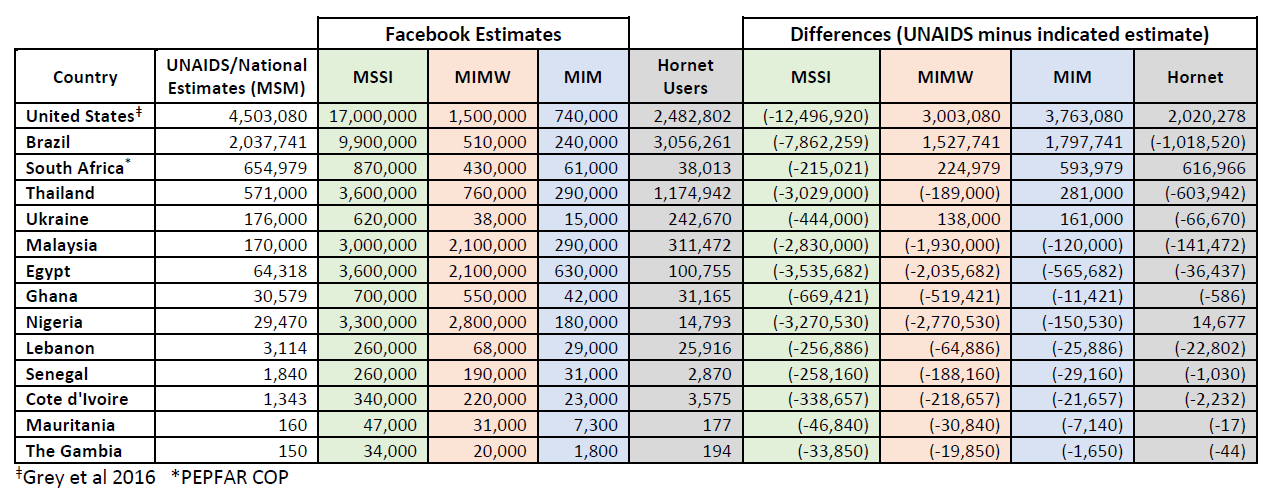

Supplement: Multimedia Appendix 5 [file publichealth_v4i1e15_app5.png]

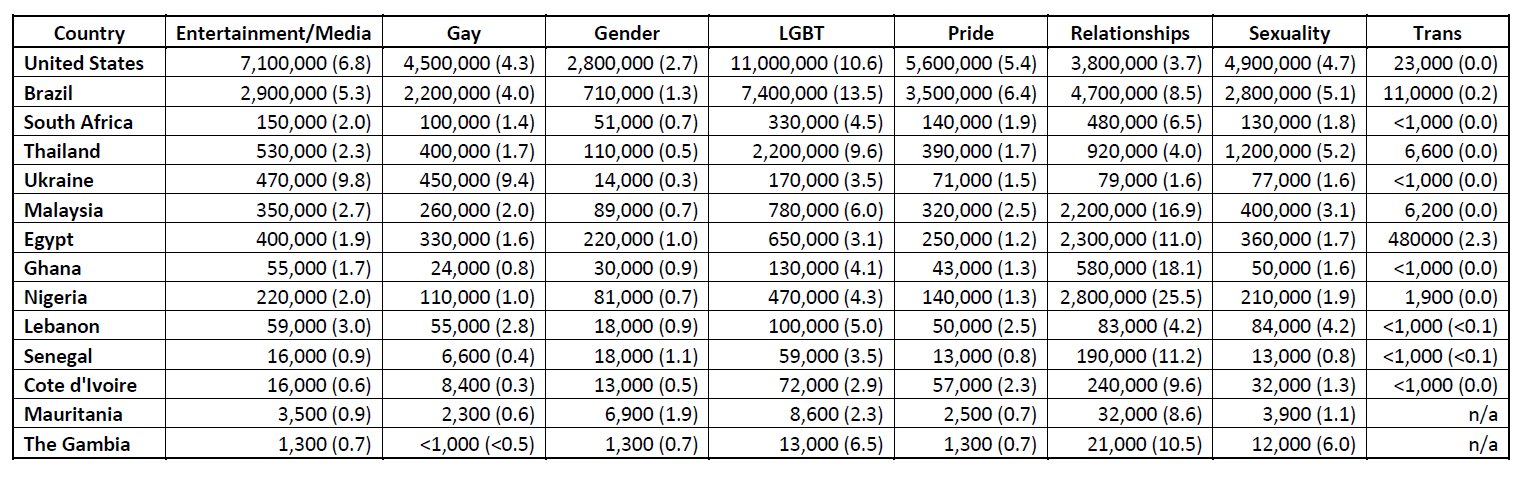

Supplement: Multimedia Appendix 6 [file publichealth_v4i1e15_app6.png]
