# Supplementary material for: Population Size Estimation of Gay and Bisexual Men and Other Men Who Have Sex With Men Using Social Media-Based Platforms
Source: JMIR Public Health Surveill. 2018 Feb 8;4(1):e15. doi: 10.2196/publichealth.9321 (PMC5824103; doi:10.2196/publichealth.9321)
Supplement: Multimedia Appendix 7 [file publichealth_v4i1e15_app7.pdf]

Age distribution of male Facebook users by country (proportion of males). Data are reported in thousands, n (%). MIM: men >18 years of age interested in men; MIMW: men >18 years of age interested in men or men and women; MSSI: men >18 years of age with at least one reported same-sex interest.

Country	Age 13-17 years			Age 18-24 years			Age 25-29 years			Age 30-34 years			Age 35+ years		
	MSSI	MIMW	MIM	MSSI	MIMW	MIM	MSSI	MIMW	MIM	MSSI	MIMW	MIM	MSSI	MIMW	MIM
United States	720 (21.2)	31 (0.9)	15 (0.4)	4600 (23.0)	320 (1.6)	190 (1.0)	3400 (21.3)	290 (1.8)	170 (1.1)	2300 (19.2)	210 (1.8)	110 (0.9)	6700 (12.4)	660 (1.2)	290 (0.5)
Brazil	830 (18.0)	24 (0.5)	21 (0.5)	4300 (25.3)	150 (0.9)	110 (0.6)	2100 (22.1)	83 (0.9)	68 (0.7)	1300 (16.9)	61 (0.8)	42 (0.5)	2200 (10.5)	210 (1.0)	87 (0.4)
South Africa	44 (5.9)	5.3 (0.7)	2.5 (0.3)	240 (10.9)	62 (2.8)	16 (0.7)	180 (12.9)	87 (6.2)	14 (1.0)	150 (12.5)	88 (7.3)	<1 (<0.1)	290 (11.6)	190 (7.6)	20 (0.8)
Thailand	350 (18.4)	32 (1.7)	31 (1.6)	1500 (17.9)	230 (2.7)	120 (1.4)	730 (17.4)	170 (4.0)	79 (1.9)	490 (14.8)	120 (3.6)	64 (1.9)	870 (11.2)	240 (3.1)	69 (0.9)
Ukraine	79 (16.8)	1.1 (0.2)	<1 (<0.2)	250 (20.8)	8.4 (0.7)	4.4 (0.4)	130 (15.7)	7.2 (0.9)	3.2 (0.4)	87 (11.0)	5.8 (0.7)	2.5 (0.3)	140 (7.0)	17 (0.9)	5.4 (0.3)
Malaysia	130 (17.3)	45 (6.0)	24 (3.2)	960 (25.3)	590 (15.5)	120 (3.2)	710 (26.3)	510 (18.9)	67 (2.5)	500 (26.3)	390 (20.5)	40 (2.1)	800 (19.0)	580 (13.8)	67 (1.6)
Ghana	25 (10.0)	9.6 (3.8)	2.3 (0.9)	260 (21.7)	170 (14.2)	19 (1.6)	180 (28.1)	150 (23.4)	9.7 (1.5)	100 (20.8)	89 (18.5)	5 (1.0)	160 (19.5)	130 (15.9)	8.1 (1.0)
Nigeria	83 (15.1)	46 (8.4)	7.7 (1.4)	880 (27.5)	670 (20.9)	60 (1.9)	790 (37.6)	680 (32.4)	39 (1.9)	540 (36.0)	480 (32.0)	24 (1.6)	1100 (31.4)	940 (26.9)	53 (1.5)
Lebanon	17 (10.0)	4.6 (2.7)	3.8 (2.2)	96 (14.8)	22 (3.4)	13 (2.0)	57 (14.3)	15 (3.8)	5.9 (1.5)	35 (12.5)	<1 (<0.4)	3.5 (1.3)	73 (11.1)	20 (3.0)	6.9 (1.0)
Senegal	12 (6.0)	3.7 (1.9)	1.6 (0.8)	99 (15.0)	65 (9.8)	14 (2.1)	65 (19.7)	50 (15.2)	7.2 (2.2)	39 (13.0)	29 (9.7)	4.1 (1.4)	55 (12.8)	43 (10.0)	6.1 (1.4)
Côte d'Ivoire	14 (4.7)	8.2 (2.7)	1.6 (0.5)	120 (13.6)	65 (7.4)	7.3 (0.8)	80 (17.0)	57 (12.1)	4.9 (1.0)	58 (14.5)	41 (10.3)	3.6 (0.9)	93 (13.1)	61 (8.6)	6.8 (1.0)
Mauritania	2 (6.1)	<1 (<3.0)	<1 (<3.0)	18 (12.0)	10 (6.7)	2.9 (1.9)	12 (15.6)	8.3 (10.8)	1.7 (2.2)	8.1 (11.6)	5.8 (8.3)	1.1 (1.6)	9.3 (12.6)	7.1 (9.6)	1.6 (2.2)
The Gambia	<1 (<10.0)	<1 (<10.0)	<1 (<10.0)	9.1 (13.6)	5.3 (7.9)	<1 (<1.5)	8.9 (21.7)	5.7 (13.9)	<1 (<2.4)	7 (17.9)	4.4 (11.3)	<1 (<2.6)	9.1 (17.5)	4.3 (8.3)	<1 (<1.9)
